# Relationships between minerals’ intake and blood homocysteine levels based on three machine learning methods: a large cross-sectional study

**DOI:** 10.1038/s41387-024-00293-3

**Published:** 2024-06-01

**Authors:** Jing Fan, Shaojie Liu, Lanxin Wei, Qi Zhao, Genming Zhao, Ruihua Dong, Bo Chen

**Affiliations:** 1 Key Laboratory of Public Health Safety of Ministry of Education, School of Public Health, Fudan University, Shanghai, 200032 China; 2 Department of Clinical Nutrition, the First Affiliated Hospital of Xiamen University, School of Medicine, Xiamen University, Xiamen, 361003 China

**Keywords:** Nutrition, Epidemiology

## Abstract

**Background:**

Blood homocysteine (Hcy) level has become a sensitive indicator in predicting the development of cardiovascular disease. Studies have shown an association between individual mineral intake and blood Hcy levels. The effect of mixed minerals’ intake on blood Hcy levels is unknown.

**Methods:**

Data were obtained from the baseline survey data of the Shanghai Suburban Adult Cohort and Biobank(SSACB) in 2016. A total of 38273 participants aged 20–74 years met our inclusion and exclusion criteria. Food frequency questionnaire (FFQ) was used to calculate the intake of 10 minerals (calcium, potassium, magnesium, sodium, iron, zinc, selenium, phosphorus, copper and manganese). Measuring the concentration of Hcy in the morning fasting blood sample. Traditional regression models were used to assess the relationship between individual minerals’ intake and blood Hcy levels. Three machine learning models (WQS, Qg-comp, and BKMR) were used to the relationship between mixed minerals’ intake and blood Hcy levels, distinguishing the individual effects of each mineral and determining their respective weights in the joint effect.

**Results:**

Traditional regression model showed that higher intake of calcium, phosphorus, potassium, magnesium, iron, zinc, copper, and manganese was associated with lower blood Hcy levels. Both Qg-comp and BKMR results consistently indicate that higher intake of mixed minerals is associated with lower blood Hcy levels. Calcium exhibits the highest weight in the joint effect in the WQS model. In Qg-comp, iron has the highest positive weight, while manganese has the highest negative weight. The BKMR results of the subsample after 10,000 iterations showed that except for sodium, all nine minerals had the high weights in the joint effect on the effect of blood Hcy levels.

**Conclusion:**

Overall, higher mixed mineral’s intake was associated with lower blood Hcy levels, and each mineral contributed differently to the joint effect. Future studies are available to further explore the mechanisms underlying this association, and the potential impact of mixed minerals’ intake on other health indicators needs to be further investigated. These efforts will help provide additional insights to deepen our understanding of mixed minerals and their potential role in health maintenance.

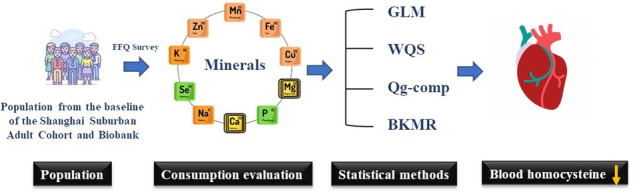

## Introduction

Homocysteine (Hcy), a sulfurous amino acid, cannot be synthesized in the body but can only be converted from methionine [1]. When the metabolic process of methionine is blocked in body, blood Hcy levels will be a progressive increase, resulting in the occurrence of hyperhomocysteinemia (hHcy), which was diagnosed clinically as a blood Hcy concentrations of more than 15 μmol/L [2]. Increasing Hcy in body can put a wide range of diseases at increased risk of development and progression by hypomethylating deoxyribonucleic acid (DNA) [3], increasing oxidative stress [4], and promoting cellular damage and apoptosis [5]. A recent meta-analysis showed that the estimated prevalence of hHcy in China was 37.2%, with a gradual increasing trend [6]. Hcy metabolism is regulated by multiple nutrients. Inadequate nutrient intake from dietary imbalance is one of the causes of abnormal Hcy metabolism [7]. Most nutritional studies on Hcy primarily emphasize B vitamins, including vitamin B_6_, B_12_, and folic acid [8, 9]. Lower levels of these vitamins contribute to elevated Hcy by impeding the methylation process, hindering the conversion of Hcy to methionine, and ultimately resulting in the aggregation of Hcy [1, 8, 9]. Concurrently, a limited number of studies have sought to investigate the correlation between blood Hcy levels and nutrients beyond B vitamins, including minerals [10, 11]. Only two publications have scrutinized the link between dietary calcium intake and serum Hcy levels, and the association was negative. Interestingly, this negative association retained its significance even after adjusting for social characteristics, lifestyle factors, lipid levels, and other blood parameters [10, 11]. Nevertheless, the scope of both studies was limited to postmenopausal women, and thus, the association between dietary calcium intake and serum Hcy levels remains unknown in other adult populations. In a case-control study involving 203 subjects without significant organic disease, a positive correlation emerged between the risk of developing hHcy and plasma zinc concentration and, there was a significant reduction in the risk of hHcy development when the plasma zinc concentration fell below 83.89 μmol/L [12]. Another study examined blood selenium levels and revealed a significant non-linear relationship with hHcy prevalence, where participants in the upper three blood selenium quartiles had a lower risk of hHcy compared to the lowest quartile of blood selenium concentrations [13]. In summary, the relationship between minerals and hHcy does exist, and this can be explained through the metabolic pathways of Hcy. Minerals play integral roles in intricate physiological processes within the body, serving as constituents of diverse metabolic enzymes or acting as cofactors that regulate enzyme activity. Notably, they contribute to the functioning of pivotal enzymes such as Paraoxonase 1 and Betaine-Hcy methyltransferase, crucial players in the metabolism of homocysteine [14, 15]. However, individuals consistently obtain various minerals simultaneously from a diverse range of foods, and the initiation of mineral effects in the human body is nearly simultaneous. There may be synergistic or antagonistic interactions among minerals, making the overall impact on blood Hcy levels unknown. Additionally, the specific weight of each mineral in the overall impact remains uncertain.

Machine learning has become integral across various domains, including environmental health. In scenarios where the assessment involves combined exposures to multiple pollutants, conventional models such as multiple linear regression and multivariate logistic regression may be deemed inaccurate when attempting to directly incorporate one of the exposures [16–18]. The nutrition field also requires the involvement of machine learning methods to complement traditional statistical methods to draw more scientific results. Scholars have initiated the application of machine learning techniques to investigate the intricate association between the combined consumption of diverse nutrients and subsequent health outcomes, albeit with limited coverage in existing literature [18, 19]. Unfortunately, investigations into the correlation between the combined intake of various minerals and hHcy are lacking in the existing body of research. Therefore, based on the current scientific evidence, we hypothesize that a mixed intake of multiple minerals may be associated with a reduction in hHcy.

There are a total of 21 mineral elements in three categories that are essential for constituting human tissues, participating in body metabolism and maintaining physiological functions [20]. The top 10 minerals (calcium, phosphorus, sodium, potassium, magnesium, iron, copper, zinc, selenium and manganese) are found in relatively high levels in the human body, and there has been extensive research on their association with various diseases. What’s more, their contents in food were adequately detected in the Chinese Food Composition Table (CFCT). Thus. We included these 10 minerals in this study. In China, around 181.3 million residents aged 40 and older have hHcy, with a prevalence of 7.4% in Shanghai. In Shanghai, there was an established large-scale on-site investigation named Shanghai Suburban Adult Cohort and Biobank (SSACB). We enrolled 38,273 participants from the baseline survey of SSACB and used the emerging machine learning methods to explore the relationship between multiple those ten minerals’ intake and hHcy, to identify the joint effect and the contribution of different minerals.

## Materials and methods

### Study population

**Fig. 1:**
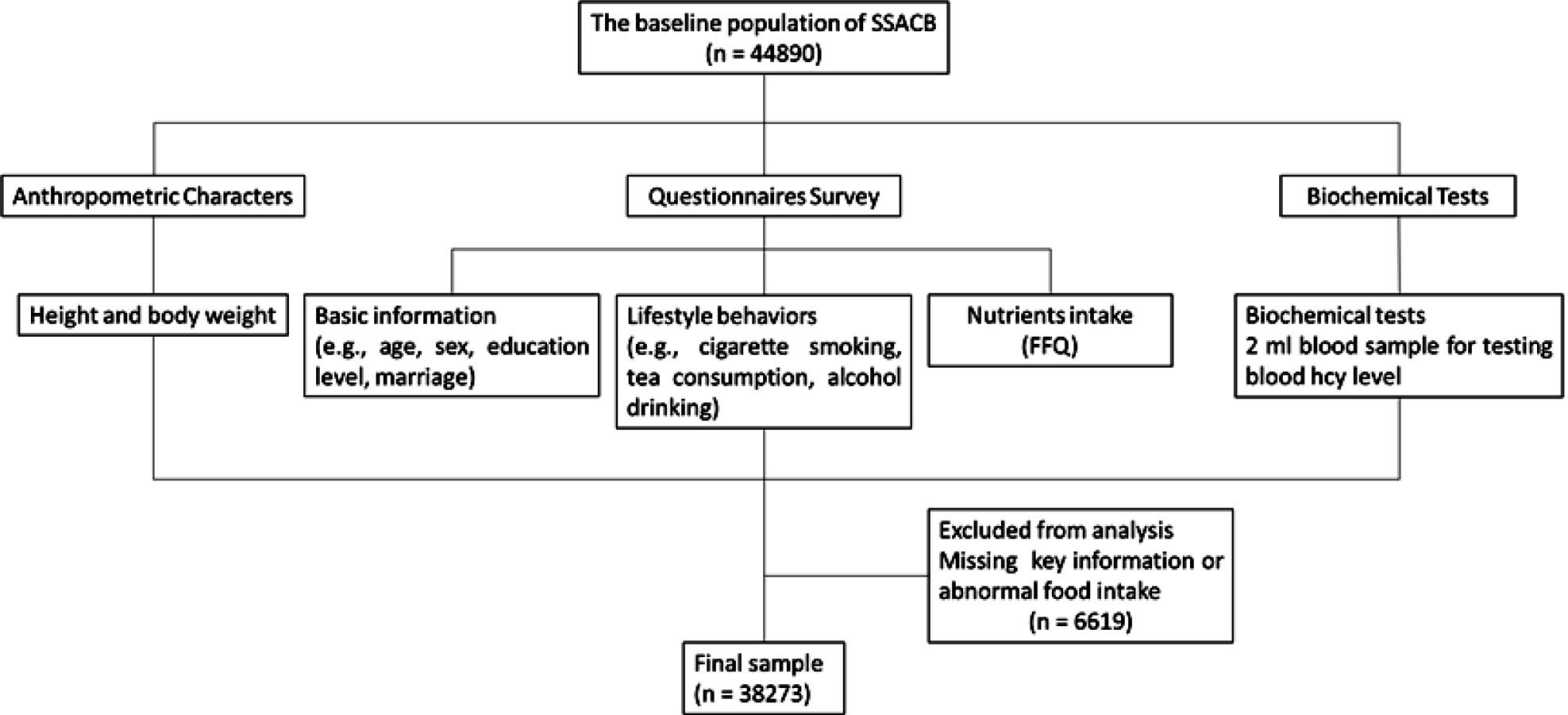
Study flowchart.

Data were obtained from the baseline survey data of the SSACB in 2016 [21]. Participants was recruited from two districts in Shanghai: Songjiang and Jiading districts. Four communities in each district were selected based on economic and population size, and one-third of the neighborhood committees (villages) in each community were randomly selected as cohort sites. Questionnaire survey, food frequency questionnaire (FFQ), physical examinations and biological specimens were completed by standardized trained investigators. More details on sampling and survey methodology were described in the previous study [21]. A total of 44,890 participants aged 20–74 years living permanently were included. Individuals were excluded those with missing FFQ or energy intakes less than 800 kcal/d (3349.4 kJ/d) or more than 4000 kcal/d (16,747.2 kJ/d) for males and less than 500 kcal/d (2093.4 kJ/d) or more than 3500 kcal/d (14,653.8 kJ/d) for females, or those with missing key information [22]. Finally, 38,273 eligible individuals were effectively enrolled as the participants of this study. The flowchart of this study was showed in Fig. 1.

This study was ethically reviewed by the Ethics Committee of the School of Public Health, Fudan University (Grant Number: 2016-04-0586) and, all participants signed the informed consent form prior to the survey. All procedures were conducted according to the guidelines in the Declaration of Helsinki.

### Assessment of the minerals’ intake

Food intakes were investigated by well-trained investigators through face-to-face interviews using a standard FFQ containing 29 food groups, including rice and cereal products and various other food types. The reliability and validity of the FFQ has previously been demonstrated, briefly, the results demonstrated a correlation coefficient for both food groups (reliability: 0.36–0.54; validity: 0.20–0.41) and nutrients (reliability: 0.39–0.60; validity: 0.12–0.42). The food data acquired through the FFQ were integrated with the second edition of the CFCT. Subsequently, the final consumption of the ten minerals and energy intake was computed based on food weights [23]. These minerals include calcium, phosphorus, sodium, potassium, magnesium, iron, copper, zinc, selenium, and manganese. Each mineral’s intake was log-transformed to acquire the normal distribution and included in all models for analysis.

### Measurement of biological specimens

Fasting venous blood was collected from all participants in the morning of the investigation day and blood Hcy concentrations were measured by fluorescence chromatography. According to the diagnostic criteria [2], the individuals with blood Hcy concentrations ≥15 μmol/L were defined as hHcy. Blood Hcy concentrations were included in analyses as log-transformed continuous variables and hHcy was included as a dichotomous variable (1 = hHcy, 0 = non- hHcy).

### Covariates

We employed Directed Acyclic Graph (DAG), a theory-driven variable screening method, to select the appropriate covariates to enter the model. For more details, see Fig. S1. Alcohol and tea drinking were categorized as yes(ever had at least 3 drinks/tea per week for more than 6 months) and no. Cigarette smoking consisted of three categories: “smoking” was defined as smoking >1 cigarette per day for ≥6 months continuously or cumulatively and still smoking at the time of the survey; “smoking cessation” was defined as ever smoking and not smoking at the time of the survey; “never smoking” was defined as never smoked previously. Marriage was classified as married, divorced, widowed and unmarried. Educational levels were classified as primary school and below, junior high School, senior high school, and university and above. Physical activity was calculated as metabolic equivalent (MET) values based on physical activity intensity, duration and frequency of each activity, and MET values were categorized into four levels according to quartiles. In addition, energy intake (Kcal) was obtained by FFQ and the second edition of the CFCT. Height and weight were measured using the national standard WS/T424-2013 [24] with height accurate to 0.1 cm and weight accurate to 0.1 kg, and the measurements were repeated three times to take the average. Finally, body mass index (BMI) was calculated according to the formula: Weight (kg)/Height^2^ (m^2^), with BMI < 18.5 kg/m² being underweight, 18.5–23.9 kg/m² being normal, 23.9–27.9 kg/m² being overweight and over 28 kg/m² being obese [25]. Health condition was obtained from questionnaire by asking whether have hypertension, coronary heart disease or diabetes or not. In addition, considering the effects of vitamin B_6_ and vitamin B_12_ on blood hcy levels, the intake of these two nutrients was also included as covariates. The above variables were used as covariates in each statistical model to adjust for their potential effect on the relationship between minerals and blood Hcy levels.

### Statistical analysis

The statistical analysis in this study consisted of three parts. Firstly, all participants were divided into hHcy and non-hHcy groups, and the description of characteristics between the two groups was presented as mean ± standard deviation (SD) for continuous variables and as frequency (proportion) for categorical variables; differences in characteristics between the two groups were compared using a student *t*-test for continuous variables and a chi-square test for categorical variables. Secondly, blood Hcy levels were included in the models as continuous and categorical variables, respectively, and used the multiple linear regression and multivariate logistic regression to identify the single effect of each mineral on blood Hcy levels. To reduce the false discovery rate and avoid type I errors, adjusted *p*-values were evaluated using the Benjamini-Hochberg procedure [26]. Third, three machine learning-based methods, including WQS, Qg-comp regression and BKMR, were used to explore the overall effect of these ten minerals on blood Hcy levels and the weights of each mineral. The BKMR method is not applicable to extremely large populations, and operational efficiency is reduced to a greater extent when the number of observations exceeds 1000, even requiring lengths of time ranging from several days to several weeks. Gender and age are immutable influences on hHcy. Therefore, we included a 10% proportion of subsamples from the total sample population [27] stratified by hHcy, gender, and age group in the BKMR analysis. Sampling was performed in SAS 9.4 software using PROC SURVEYSELECT. All models in the current study were corrected for the potential effects of sex, age, education levels, marriage, cigarette smoking, alcohol and tea drinking, energy intake, physical activity, BMI, health condition and the intake of Vitamin B_6_ and B_12_. Meanwhile, The intakes of the ten minerals were logarithmically included in the model. All data cleaning and statistical analysis procedures were performed in SAS (version 9.4) and R software (version 4.3.2). *P* < 0.05 was considered statistically significant. The updated R packages and instructions for usage are available in Git Hub.

### The respective advantages of three machine learning-based methods

#### The Weighted Quantile Sum (WQS) regression model

WQS is a useful tool for addressing multiple substances, especially when they are highly correlated with each other, and the method can produce results with good interpretation for the purpose of screening for high-weighted influences [28, 29]. WQS constructs a weighted index in a supervised manner to assess the overall effect of the various substances and the contribution of each component of the mixture to the overall effect. The training dataset and validation dataset were in the ratio of 4:6. Individual weights of more than 0.1 (1/10) for each mineral were considered to have a significant effect on the total index [18].

#### Quantile G-computation (Qg-comp)

In this study, we used the Qg-comp.boot function to verify the linearity of the effect of cumulative intake and the Qg-comp.noboot function to construct a linear model with a built-in Bayesian algorithm by dividing all the mineral intakes into quintiles, giving positive and negative weights to each mineral intake. Minerals with individual weights greater than 0.05 were considered to have a significant effect on Qg-comp scores.

#### Bayesian kernel machine regression (BKMR) model

BKMR does not require setting parameter expressions, allowing for non-linear effects and interactions, and can generate kernel functions based on the mixture variables put into the model, and then use Bayesian sampling and analysis to generate dose- response curves for the mixture components and the outcome variables [30]. Similar to WQS and Qg-comp, BKMR produces a similar weighting of individual substances in the overall effect, known in its model as the posterior inclusion probability (PIP), where a larger value of PIP implies a higher relative importance of the substance [31]. The PIP cutoff value was set to 0.5 for determining whether mineral intake was important. Finally, we calculated the total effect of these ten minerals’ intakes on blood hcy levels, as well as possible interactions between each mineral intake.

## Results

### Population characteristics

In this study, 38,273 eligible participants were enrolled including 13,276 males and 24,547 females. The prevalence of hHcy was 21.6%. As shown in Table 1, the average age was 55.9 ± 11.1 years, with a predominance of the age range 45 to 65 years (61.5%). The prevalence of hHcy was higher in the older age groups, male, lower education levels, unmarried, smokers, alcohol and tea drinkers, less physically active and those with hypertension and diabetes, all with statistically significant differences (*P* < 0.05). Participants with hHcy had higher energy intake and higher BMI and were more likely to be overweight and obesity. Minerals’ intake of the participants was presented in Table S1.

**Table 1:** Basic information of the participants.

Variables	Total (*n* = 38,273)	non-hHcy (*n* = 30,016, 78.4%)	hHcy (*n* = 8257, 21.6%)	*P* value
Age (years)	55.9 ± 11.1	55.5 ± 11.1	57.4 ± 11.1	<0.001
Age groups				<0.001
20≤Age<45	5688 (14.9)	4691 (82.5)	997 (17.5)	
45≤Age<65	23,545 (61.5)	18,668 (79.3)	4877 (20.7)	
Age≥65	9040 (23.6)	6657 (73.6)	2383 (26.4)	
Sex				<0.001
Male	13,726 (32.9)	8613 (62.8)	5113 (37.2)	
Female	24,547 (64.1)	21,403 (87.2)	3144 (12.8)	
Education levels				<0.001
Primary school and below	15,560 (40.9)	12,233 (78.1)	3427 (21.9)	
Junior high School	14,966 (39.1)	11,662 (77.9)	3304 (22.1)	
Senior high School	4883 (12.8)	3839 (78.6)	1044 (21.4)	
University and above	2764 (7.2)	2282 (82.6)	482 (17.4)	
Marriage				0.038
Married	35,568 (92.9)	27,911 (78.5)	7657 (21.5)	
Divorced	571 (1.5)	470 (82.3)	101 (17.7)	
Widowed	1659 (4.3)	1296 (78.1)	363 (21.9)	
Unmarried	475 (1.2)	339 (71.4)	136 (28.6)	
Cigarette smoking				<0.001
Never smoking	29,962 (78.3)	24,877 (83.0)	5085 (17.0)	
Smoking cessation	1269 (3.3)	825 (65.0)	444 (35.0)	
Smoking	7042 (18.4)	4314 (61.3)	2728 (38.7)	
Alcohol drinking				<0.001
Drinkers	4525 (11.8)	2937 (64.9)	1588 (35.1)	
Non-drinkers	33,748 (88.2)	27,079 (80.2)	6669 (19.8)	
Tea drink				<0.001
Drinkers	27435 (71.7)	22446 (81.8)	4989 (18.2)	
Non-drinkers	10838 (28.3)	7570 (69.9)	3268 (30.1)	
Physical activity				<0.001
Quantile 1	4742 (12.4)	3291 (69.4)	1451 (30.6)	
Quantile 2	18059 (47.2)	13,958 (77.3)	4101 (22.7)	
Quantile 3	14619 (38.2)	12,086 (82.7)	2553 (17.3)	
Quantile 4	853 (2.2)	681 (79.8)	172 (20.2)	
Energy intake (kcal)	1187.8 ± 477.4	1169.7 ± 467.7	1253.6 ± 505.5	<0.001
BMI (kg/m²)	24.2 ± 3.4	24.2 ± 3.4	24.5 ± 3.4	<0.001
BMI status				<0.001
Underweight	1146 (3.0)	938 (81.9)	208 (18.1)	
Normal weight	17,802 (46.5)	14,286 (80.3)	3516 (19.8)	
Overweight	14,531 (38.0)	11,105 (76.4)	3426 (23.6)	
Obesity	4794 (12.5)	3687 (76.9)	1107 (23.1)	
Hypertension				<0.001
Yes	12514 (32.7)	9411 (75.2)	3123 (24.8)	
No	25759 (67.3)	20605 (80.0)	5154 (20.0)	
Coronary heart disease				0.724
Yes	1550 (4.1)	1210 (78.1)	340 (21.9)	
No	36723 (95.9)	28806 (78.4)	7917 (21.6)	
Diabetes				0.018
Yes	2914 (7.6)	2336 (80.2)	578 (19.8)	
No	35359 (92.4)	27680 (78.3)	7679 (21.7)	
VitaminB_6_ intake	1.1 (0.8, 1.5)	1.1 (0.8, 1.5)	1.1 (0.8, 1.5)	<0.001
VitaminB_12_ intake	3.2 (2.0, 5.3)	3.2 (2.0, 5.3)	3.3 (2.0, 5.5)	<0.001
Homocysteine (μmol/L)	13.6 ± 11.9	11.4 ± 2.4	21.7 ± 23.5	<0.001

The data was exhibited by Mean ± SD and Percentage (frequency).

*hHcy* Hyperhomocysteinemia, *BMI* body mass index.

### Associations between minerals’ intake and hHcy risk: The single effect

As shown in Table 2, we employed multiple linear regression and multivariate logistic regression models to explore the associations between minerals’ intake and blood Hcy levels or the risk of hHcy, adjusted by age, sex, education levels, marriage, cigarette smoking, alcohol drinking, tea drinking, energy intake, physical activity, BMI, hypertension, coronary heart disease, diabetes, and the intake of Vb_6_ and Vb_12_. The results of multiple linear regression showed that higher intake of calcium, phosphorus, potassium, magnesium, iron, zinc, copper and manganese were associated with lower levels of blood Hcy levels (*P* < 0.05); the results of multivariate logistic regression showed that higher intake of calcium, phosphorus, potassium, magnesium, iron, zinc, selenium and copper were associated with lower hHcy prevalence, with all differences being statistically significant.

**Table 2:** Results of multiple linear and multivariate logistic regression of each mineral in relation to blood Hcy levels/hHcy risk.

Minerals	*β* (95%CI)	Adjusted *P* value	*OR* (95%*CI*)	Adjusted *P* value
Calcium	−0.748 (−0.103, −0.393)	0.0002	0.793 (0.734, 0.858)	0.0002
Phosphorus	−1.624 (−2.383, −0.865)	0.0002	0.538 (0.454, 0.637)	0.0002
Potassium	−1.105 (−1.652, −0.557)	0.0002	0.741 (0.658, 0.835)	0.0002
Sodium	−0.163 (−0.404, −0.078)	0.2047	0.992 (0.941, 1.045)	0.7570
Magnesium	−1.708 (−2.409, −1.007)	0.0002	0.682 (0.585, 0.795)	0.0002
Iron	−1.068 (−1.818, −0.318)	0.0073	0.812 (0.689, 0.958)	0.0173
Zinc	−1.729 (−2.554, −0.904)	0.0002	0.579 (0.483, 0.695)	0.0002
Selenium	−0.248 (−0.717, 0.221)	0.2999	0.827 (0.746, 0.916)	0.0004
Copper	−0.498 (−0.852, −0.144)	0.0073	0.851 (0.786, 0.920)	0.0002
Manganese	−1.080 (−1.701, −0.458)	0.0012	0.886 (0.774, 1.014)	0.0881

All models were adjusted by age, sex, education levels, marriage, cigarette smoking, alcohol drinking, tea drinking, energy intake, physical activity, BMI, hypertension, coronary heart disease, diabetes, and the intake of Vb_6_ and Vb_12_.

*hHcy* Hyperhomocysteinemia.

*P* value were adjusted by Benjamini–Hochberg Method.

### Associations of minerals’ intake with hHcy risk: WQS model

**Fig. 2:**
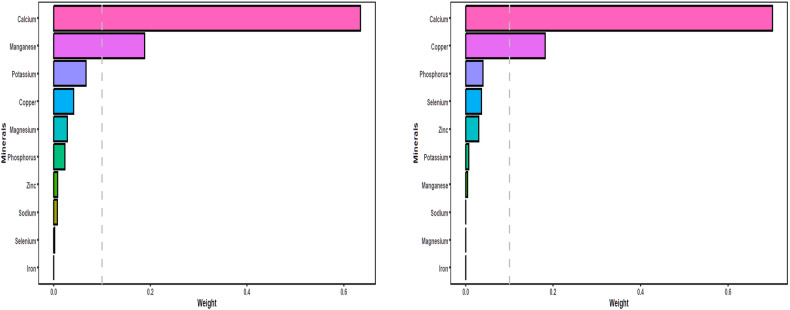
WQS model regression index weights for homocysteine (figure left) and hHcy (figure right). The WQS models were adjusted by age, sex, education levels, marriage, cigarette smoking, alcohol drinking, tea drinking, energy intake, physical activity, BMI, hypertension, coronary heart disease, diabetes, and the intake of Vb_6_ and Vb_12_. WQS weighted quantile sum, hHcy Hyperhomocysteinemia.

The overall effect of minerals on Hcy levels and hHcy prevalence were obtained through the WQS model, as shown in Table 3. After adjusting for all covariates, WQS model showed that the overall effect of these minerals was negatively associated with blood Hcy levels (WQS: −0.201, *P* = 0.001) and the prevalence of hHcy (WQS: –0.074, *P* < 0.002). Figure 2 illustrates the weight of each mineral in the overall effect using the WQS model, with calcium contributing most to blood Hcy levels and hHcy risk (Hcy level: mean weight = 0.701, hHcy risk: mean weight = 0.634). Additionally, magnesium emerged as another significant mineral associated with higher blood Hcy levels, while copper was linked to increased hHcy risk. The weights of each mineral were shown in Table S2.

**Table 3:** Overall association of mixed minerals on Hcy and hHcy obtained by WQS model.

Model	Outcomes	Estimate	Std. Error	*P* value
WQS	Hcy	−0.201	0.062	0.001
	hHcy	−0.074	0.023	0.002

All models were adjusted by age, sex, education levels, marriage, cigarette smoking, alcohol drinking, tea drinking, energy intake, physical activity, BMI, hypertension, coronary heart disease, diabetes, and the intake of Vb_6_ and Vb_12_.

*WQS* weighted quantile sum, *Hcy* homocysteine, *hHcy* Hyperhomocysteinemia.

### Associations of minerals’ intake with hHcy risk: Qg-comp model

**Fig. 3:**
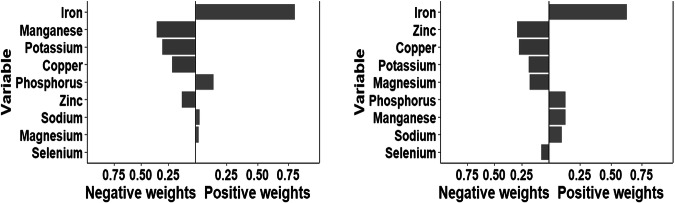
Qg-comp model regression index weights and joint effect (95% CI) for Hcy (figure left) and hHcy (figure right). The Qg-comp models were adjusted by age, sex, education levels, marriage, cigarette smoking, alcohol drinking, tea drinking, energy intake, physical activity, BMI, hypertension, coronary heart disease, diabetes, and the intake of Vb_6_ and Vb_12_. Qg-comp Quantile g-computation, hHcy Hyperhomocysteinemia.

**Fig. 4:**
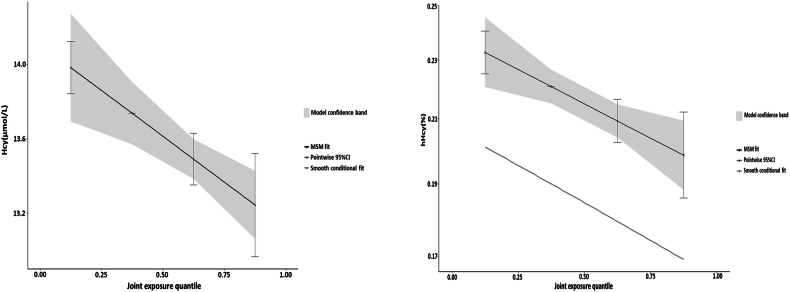
Qg-comp regression analysis of the relationship between minerals mixture and blood Hcy level (left) and hHcy risk(right). The Qg-comp models were adjusted by age, sex, education levels, marriage, cigarette smoking, alcohol drinking, tea drinking, energy intake, physical activity, BMI, hypertension, coronary heart disease, diabetes, and the intake of Vb_6_ and Vb_12_. Qg-comp Quantile g-computation, hHcy Hyperhomocysteinemia.

As shown in Table 4 and Fig. 3, Qg-comp model revealed that the increases in each quartile of mixed mineral levels were associated with a decrease in Hcy levels (qg-comp: −0.024, *P* = 0.8874) and the lower hHcy prevalence (qg-comp: −0.006, *P* = 0.8453). For hHcy prevalence risk, the highest effect of mixed minerals was iron with a weight value of 0.628 in the positive direction, and for blood Hcy levels, the highest effect of mixed minerals was iron with a weight value of 0.8009 in the positive direction, and the highest negative weight was magnesium with a weight value of 0.3553 (Fig. 4).

**Table 4:** Overall association of mixed minerals on Hcy and hHcy obtained by Qg-comp model.

Model	Outcomes	Estimate	Std. Error	*P* value
Qg-comp	Hcy	−0.024	0.137	0.8874
	hHcy	−0.006	0.030	0.8453

All models were adjusted by age, sex, education levels, marriage, cigarette smoking, alcohol drinking, tea drinking, energy intake, physical activity and BMI, hypertension, coronary heart disease, diabetes, and the intake of Vb_6_ and Vb_12_.

*Hcy* homocysteine, *hHcy* Hyperhomocysteinemia.

### Associations of minerals’ intake with hHcy risk: BKMR models

**Fig. 5:**
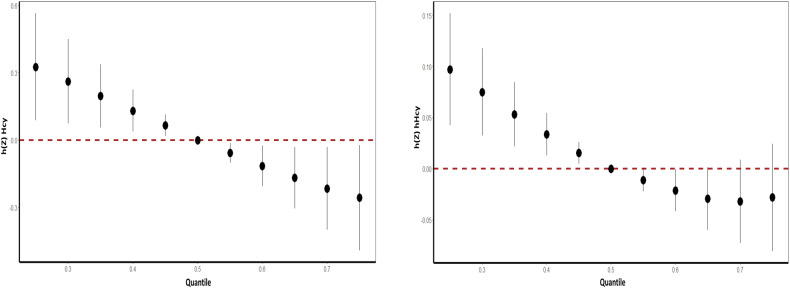
Associations between mixed minerals and Hcy (figure left) and hHcy (figure right) obtained by BKMR model. The BKMR models were adjusted by age, sex, education levels, marriage, cigarette smoking, alcohol drinking, tea drinking, energy intake, physical activity, BMI, hypertension, coronary heart disease, diabetes, and the intake of Vb_6_ and Vb_12_. BKMR Bayesian kernel machine regression, Hcy homocysteine, hHcy Hyperhomocysteinemia.

**Fig. 6:**
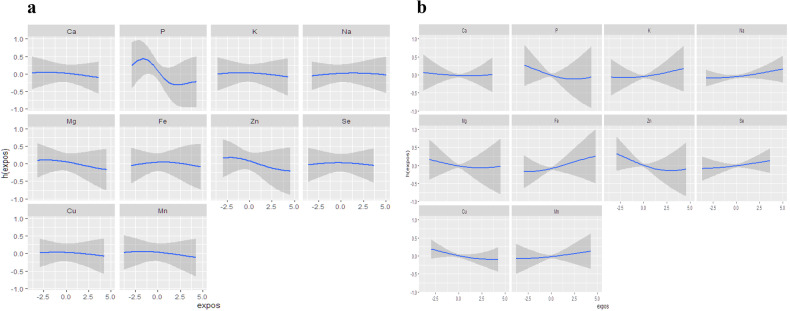
Exposure-response relationship of minerals and blood Hcy levels based on BKMR. Univariate exposure–response functions and 95% confidence interval for each mineral on the effect of Hcy (**a**) and hHcy (**b**), with other minerals fixed at the median. The BKMR models were adjusted by age, sex, education levels, marriage, cigarette smoking, alcohol drinking, tea drinking, energy intake, physical activity, BMI, hypertension, coronary heart disease, diabetes, and the intake of Vb6 and Vb12. BKMR Bayesian kernel machine regression, Hcy homocysteine, hHcy hyperhomocysteinemia.

The BKMR produced an overall association of mixed minerals with Hcy and hHcy, as shown in Fig. 5 with all mixed minerals at the 50% percentile or higher, blood Hcy levels and hHcy prevalence decreased, indicating that mixed minerals were negatively associated with the risk of hHcy, with all differences were statistically significant. Similar to the WQS and qg-comp models, the BKMR model also calculated the contribution of each mineral on the effect of Hcy and hHcy, also known as the PIP, with higher PIP indicating greater importance for outcome. As shown in Table 5, the highest contributor to blood Hcy levels was phosphorus (PIP = 0.7232), followed by zinc, manganese, magnesium, calcium, selenium, potassium and iron; the highest contributor to the prevalence of hHcy was zinc (PIP = 0.6904), followed by phosphorus, iron, potassium, copper and magnesium. Figure 6a showed that calcium, phosphorus, potassium, magnesium, zinc, copper and manganese intake were negatively associated with higher level of blood Hcy, and Fig. 6b showed that calcium, phosphorus, magnesium, zinc and cooper intake were negatively associated with higher risk of hHcy when other minerals were taken as median values although there were no statistically significant differences. Furthermore, the interaction results showed certain interactions between several minerals other than zinc and phosphorus in their effects on the risk of hHcy prevalence, which were not reflected in the changes in blood Hcy concentrations (Fig. S2).

**Table 5:** Posterior inclusion probability (PIP) of the relationship between minerals and Hcy and hHcy.

Minerals	PIP (Hcy)	PIP (hHcy)
Calcium	0.5928	0.4878
Phosphorus	0.7232	0.6802
Potassium	0.5436	0.5838
Sodium	0.4574	0.4252
Magnesium	0.6082	0.5034
Iron	0.5290	0.6106
Zinc	0.6574	0.6904
Selenium	0.5832	0.4252
Copper	0.4968	0.5442
Manganese	0.6156	0.4280

PIP indicates the relative importance of the impact on the outcome, with higher indicating greater importance to the outcome.

*PIP* posterior inclusion probability, *Hcy* homocysteine, *hHcy* Hyperhomocysteinemia.

## Discussion

The current study confirms the initial hypothesis that mixed mineral intake is associated with a lower risk of hHcy. First, results from traditional multiple linear regression showing that eight minerals were associated with lower blood Hcy concentrations, as well as results from traditional multivariate logistic regression showing that eight minerals were associated with lower hHcy risk. Then, using three innovative machine learning methods, a mixed mineral intake was found to be associated with lower blood hcy concentrations and lower hHcy risk. Anyway, a growing body of literature focuses on the relationship between nutrients and health indicators, and the present study emphasizes the relationship between minerals in nutrients and hHcy risk.

In the traditional multifactorial model, the eight minerals associated with lower blood Hcy concentrations were calcium, phosphorus, potassium, magnesium, iron, zinc, copper, and manganese, whereas the eight minerals associated with lower hHcy risk were calcium, phosphorus, potassium, magnesium, iron, zinc, selenium, and copper. Traditional multifactorial models have been widely used in previous studies, with ease of data processing and ease of interpretability of results being the primary reasons. However, it is not appropriate to include each mineral simultaneously in one model because of the high correlation among these minerals. Therefore, the inclusion of only a single mineral in a model without considering the effects of the other minerals may cause the appearance of false positives or false negatives, with less confidence in the results. Furthermore, nonlinearities and interactions among minerals may exist, and the joint effect of mixed minerals on hHcy risk is not available in traditional models, with innovative methodologies needing to be used in this field as soon as possible.

WQS and Qg-comp are novel approaches that have been used to answer questions related to mixed exposure, and they allow for reporting the joint effect as well as the weighting of each exposure in the joint effect [32]. Both WQS and Qg-comp in the current study reported a negative association between higher mixed minerals’ intake and lower hHcy risk. The minerals with the highest negative weights in the WQS model are calcium and copper, and it is manganese in the Qg-comp model. It can be found that the two novel methods show a higher sensitivity to the results relative to the traditional model, after considering the influence of other minerals.

BKMR is another kind of machine learning method to analyze the relationship between mixed exposures and health. It additionally outputs univariate effects and interactions in parallel to reporting weights for joint effects and single exposures. The results of the BKMR model in the current study suggested that higher intakes of the nine minerals, except for sodium, were associated with a lower risk of hHcy and had a high weighting in the joint effect. Univariate effects findings also indicated that higher intakes of phosphorus, zinc, copper, and magnesium were associated with lower hHcy risk when levels of each of the remaining minerals were controlled. Also, there were some interactions between the ten minerals. Thus, the novel methods do more analyzing and interpreting on the data relative to the traditional models, elucidating the complex relationship between the mixed minerals and hHcy.

Previous studies have also used advanced statistical methods in exploring the relationship between mixed nutrients and health, despite the limited number of studies [33–40]. Li, RQ et al. [34] investigated the relationship between nutrient intake, inflammatory potential, and depressive symptoms in the elderly by using BKMR, identifying significant associations and revealing a collective impact of multiple nutrients on depression risk, with an offsetting effect between pro-inflammatory and anti-inflammatory diets. A Korean study evaluated the relationship between nutrients’ intake and MetS and its components in adults aged 19–80 years (*n* = 16807) using WQS regression, Qg-comp, and BKMR regression, and found that mixed nutrients’ intake was associated with a lower risk of MetS [33]. The results remained statistically significant after adjusting for basic social characteristics, smoking, alcohol consumption, and family history of disease. Minerals are an important group of nutrients, however, their joint role in the impact on health has not been sufficiently discussed nowadays. To the best of our knowledge, there is only one literature assessing the relationship between mixed minerals and health with advanced statistical methods [36]. It evaluated the relationship between six minerals (iodine, selenium, zinc, calcium, magnesium and iron) and maternal thyroid function in 489 pregnant women in Hangzhou, China. The results showed that mixed mineral concentrations were negatively correlated with TSH and positively correlated with FT3 and FT4, with iodine contributing the most. For the first time, the current study used three advanced statistical methods to find a negative joint association between mixed minerals and hHcy risk and to elucidate the existence of interactions among mixed minerals.

The normal metabolism of Hcy in the body typically maintains low levels. However, when Hcy metabolism is impaired, the concentration of Hcy in the blood increases, reaching a condition known as hHcy [41]. Disrupted Hcy metabolism can lead to a redox imbalance, heightened oxidative stress, endoplasmic reticulum stress, and altered DNA methylation, ultimately influencing the expression of genes associated with various diseases [42]. In the human body, Hcy is primarily converted through two pathways: the remethylation pathway and the trans-sulfuration pathway. The remethylation process involves the catalytic action of methionine synthase with its coenzyme vitamin B_12_, as well as betaine-Hcy methyltransferase. The trans-sulfurization pathway relies on cystathionine β-synthase and its coenzyme vitamin B_6_ for the production of cysteine. Factors affecting Hcy metabolism can indirectly contribute to its accumulation in the blood, resulting in hHcy.

Fortunately, some other literature has assessed the relationship between selected single minerals and hHcy and has elaborated on the possible mechanisms whereby minerals influence Hcy metabolism [14, 15, 43–45]. Elevating calcium intake was found to be associated with low hHcy risk in the multivariate regression, WQS regression, and BKMR in the current study. Paraoxonase 1 was involved in the metabolism of Hcy, which is a calcium ion-dependent enzyme [14]. Increasing calcium intake may promote Hcy metabolism through this pathway. It has also been found that blood calcium levels are positively correlated with Hcy [46], which is contrary to our conclusions and may be due to the fact that the correlation between blood calcium levels and dietary calcium intake may not be direct. The results of an 8-week zinc intervention trial in postmenopausal women implied that zinc supplementation may improve blood folate levels and reduce blood Hcy levels after zinc supplementation, and correlation analyses found a negative correlation between folate levels and blood Hcy levels after zinc supplementation [43]. The improvement of blood folate levels by a zinc intervention was also found in a study of an older Australian population (65–85 years) [47]. Betaine-Hcy methyltransferase, a zinc-containing catalase, plays an important role in the Hcy-to-methionine pathway [15], which may be explained as mechanisms by which zinc promotes Hcy metabolism. A study of an elderly Spanish population suggests that selenium in the blood may play a greater role in regulating Hcy levels [44], and similar connections were found in a British National Diet and Nutrition Survey [45]. This may be because the metabolic activity of methionine synthase is significantly reduced at low selenium levels, resulting in less Hcy methylation to methionine [44].

The current study has some distinct advantages. It is the first study to explore the relationship between the mixed minerals’ intake and blood Hcy levels, reporting the single and overall effects, and the contribution of each mineral to the joint effect. Moreover, three of the advanced machine learning methods were used, which provide a higher control over the complex interactions among minerals than traditional models, and the results are more interpretable. Meanwhile, we considered numerous covariates to adjust for in the model, and the results have a certain degree of confidence. However, there are limitations to the current study. First, this was a cross-sectional study and was unable to demonstrate the causal relationship between mixed minerals’ intake and blood Hcy levels or hHcy prevalence. Although it is widely recognized that the FFQ can reflect the dietary intake of participants in the long term, intake is similar to external exposure in risk assessment, which may not be as accurate as internal exposure, that is, blood mineral levels are unknown to us in this study. Therefore, perhaps in future studies, it may be possible to test the levels of various minerals in blood samples, first to see how the concentration of minerals in the blood relates to intake, and then to assess their relationship to blood Hcy, using a combined internal and external approach to give a more comprehensive explanation of the protective effect of minerals on blood Hcy. When considering the FFQ, it is important to acknowledge that the reliability and validity coefficients reported for the FFQ in our study were lower than desired. This might be due to the extended questionnaire entries and the lengthy recall time frames, which had made it challenging for participants to complete. Nevertheless, for a thorough evaluation of the population’s long-term dietary status, detailed food items and sufficiently lengthy recall time frames are indispensable. Additionally, sex imbalance in the current study should be mentioned. The proportion of females in the participants was higher than that of males, and the prevalence of hHcy was higher among males than females; sex bias was adjusted for as a covariate in models, but the effect on the results may have been substantial. Finally, the CFCT provides a relatively small variety of minerals, and although we have considered as many common minerals as possible, other minerals that we have not yet included may have some effect on outcomes. Overall, high intake of the ten minerals was associated with lower blood Hcy levels and lower hHcy risk, and different minerals had different weights in the joint effect.

## Conclusion

Each mineral has a protective effect on blood Hcy levels and hHcy prevalence, although the different contribution of different minerals to the total effect varies. Minerals are found in a wide variety of foods, and a well-balanced diet provides adequate amounts of minerals. This is effective in preventing and controlling blood Hcy levels and reducing hHcy prevalence.

### Supplementary information

Supplementary material

## Data Availability

The datasets generated during and/or analyzed during the current study are available from the corresponding author on reasonable request.
