# Supplementary material for: Relationships between minerals’ intake and blood homocysteine levels based on three machine learning methods: a large cross-sectional study
Source: Nutr Diabetes. 2024 Jun 1;14:36. doi: 10.1038/s41387-024-00293-3 (PMC11144190; doi:10.1038/s41387-024-00293-3)
Supplement: Supplementary file 1 — Supplementary material [file 41387_2024_293_MOESM1_ESM.docx]

**Relationships between minerals’ intake and blood homocysteine levels based on three machine learning methods: A large cross-sectional study**

Jing Fan^1,a^ and Shaojie Liu^2,a^, Lanxin Wei^1^, Qi Zhao^1^, Genming Zhao^1^, Ruihua Dong^1,^*, Bo Chen^1,^*

^1^ Key Laboratory of Public Health Safety of Ministry of Education, School of Public Health, Fudan University, Shanghai 200032, China

^2^ Department of Clinical Nutrition, the First Affiliated Hospital of Xiamen University, School of Medicine, Xiamen University, Xiamen 361003, China

^a^ These authors contributed equally to this work

* Correspondence: Ruihua Dong, Email: [ruihua_dong@fudan.edu.cn](mailto:ruihua_dong@fudan.edu.cn); Bo Chen, Email: [chenb@fudan.edu.cn](mailto:chenb@fudan.edu.cn)

**Table S1.** The intake of ten minerals by the participants.

| **Minerals** | **Total** | | | | | **hHcy=0** | | | | | **hHcy=1** | | | | |
| --- | --- | --- | --- | --- | --- | --- | --- | --- | --- | --- | --- | --- | --- | --- | --- |
|  | **mean** | **std** | **P50** | **P25** | **P75** | **mean** | **std** | **P50** | **P25** | **P75** | **mean** | **std** | **P50** | **P25** | **P75** |
| Calcium(mg) | 511.4 | 263.3 | 457.8 | 330.7 | 630.9 | 512.7 | 260.1 | 332.4 | 460.3 | 634.3 | 506.7 | 274.5 | 326.0 | 448.6 | 618.1 |
| Phosphorus(mg) | 829.6 | 344.6 | 761.2 | 593.6 | 980.8 | 822.3 | 338.8 | 587.1 | 756.3 | 974.8 | 856.4 | 363.8 | 615.5 | 781.0 | 1002.4 |
| Potassium(mg) | 1641.7 | 791.4 | 1494.6 | 1116.3 | 1981.6 | 1635.9 | 775.2 | 1116.2 | 1493.0 | 1978.8 | 1662.5 | 847.2 | 1116.7 | 1499.4 | 1992.0 |
| Sodium(mg) | 1064.3 | 809.9 | 867.5 | 590.2 | 1254.2 | 1051.0 | 787.2 | 586.0 | 860.3 | 1242.3 | 1112.7 | 885.9 | 602.5 | 888.9 | 1301.0 |
| Magnesium(mg) | 256.6 | 113.3 | 233.6 | 180.8 | 303.4 | 254.4 | 111.4 | 179.1 | 232.0 | 301.9 | 264.5 | 119.8 | 187.2 | 240.2 | 309.3 |
| Iron(mg) | 17.0 | 7.5 | 15.5 | 12.0 | 20.1 | 16.8 | 7.3 | 11.9 | 15.3 | 20.0 | 17.7 | 7.9 | 12.5 | 16.0 | 20.9 |
| Zinc(mg) | 8.6 | 3.6 | 7.9 | 6.2 | 10.1 | 8.5 | 3.5 | 6.1 | 7.8 | 10.0 | 9.1 | 3.9 | 6.5 | 8.2 | 10.6 |
| Selenium(μg) | 40.5 | 19.4 | 36.7 | 27.6 | 48.6 | 40.1 | 19.1 | 27.4 | 36.5 | 48.2 | 41.8 | 20.6 | 28.3 | 37.7 | 50.1 |
| Copper(mg) | 1.9 | 1.2 | 1.6 | 1.2 | 2.3 | 1.9 | 1.2 | 1.2 | 1.6 | 2.3 | 2.0 | 1.3 | 1.2 | 1.6 | 2.3 |
| Manganese(mg) | 3.7 | 1.5 | 3.4 | 2.7 | 4.4 | 3.6 | 1.5 | 2.6 | 3.4 | 4.3 | 3.9 | 1.6 | 2.8 | 3.6 | 4.6 |

**Table S2.** Weights for each mineral in the WQS model.

| **No.** | **Hcy** | | **hHcy** | |
| --- | --- | --- | --- | --- |
|  | **Minerals** | **Weight** | **Minerals** | **Weight** |
| 1 | Calcium | 0.634 | Calcium | 0.701 |
| 2 | Manganese | 0.188 | Copper | 0.182 |
| 3 | Potassium | 0.067 | Phosphorus | 0.040 |
| 4 | Copper | 0.041 | Selenium | 0.036 |
| 5 | Magnesium | 0.028 | Zinc | 0.030 |
| 6 | Phosphorus | 0.023 | Potassium | 0.007 |
| 7 | Zinc | 0.009 | Manganese | 0.004 |
| 8 | Sodium | 0.008 | Sodium | 0.000 |
| 9 | Selenium | 0.002 | Magnesium | 0.000 |
| 10 | Iron | 0.000 | Iron | 0.000 |


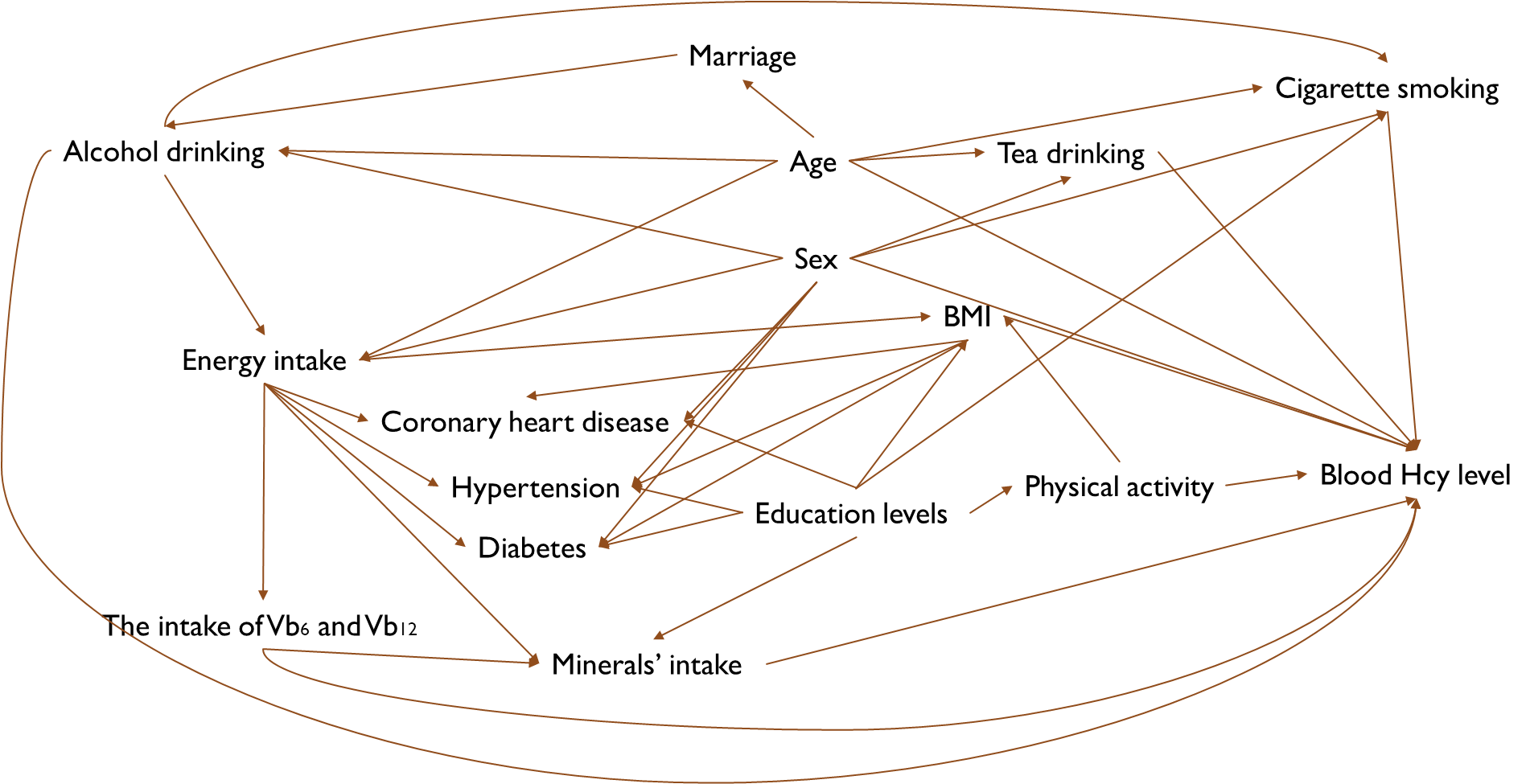


**Figure S1.** Directed Acyclic Graph (DAG) for the association between minerals intake and blood Hcy level.


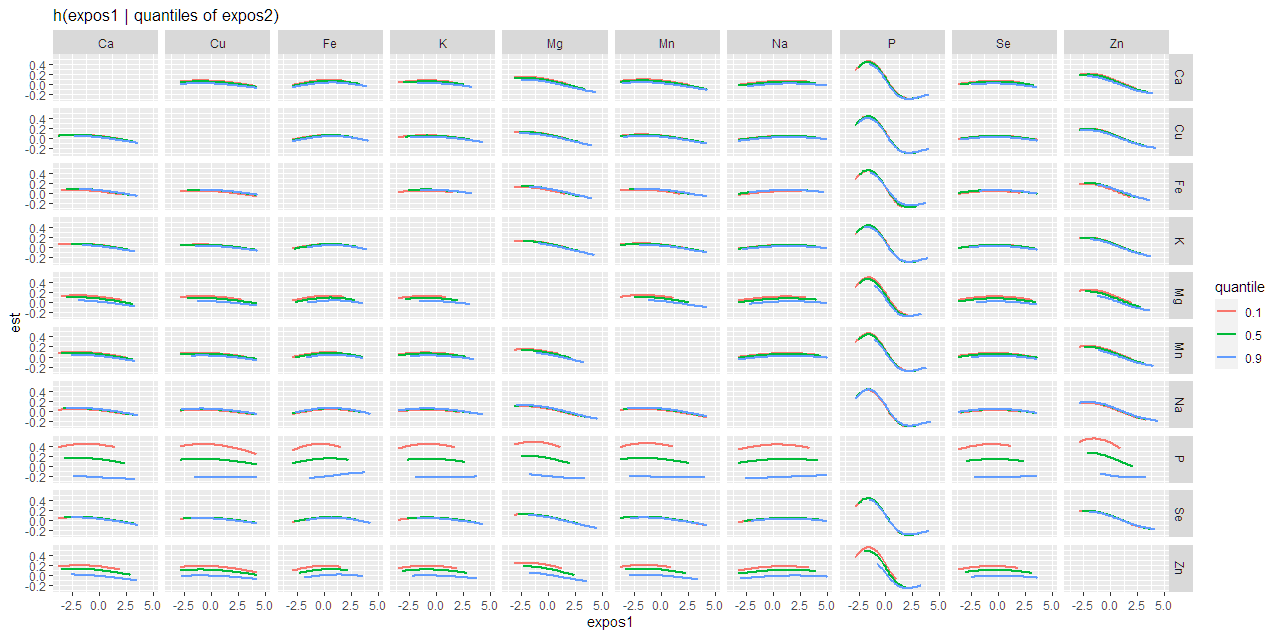

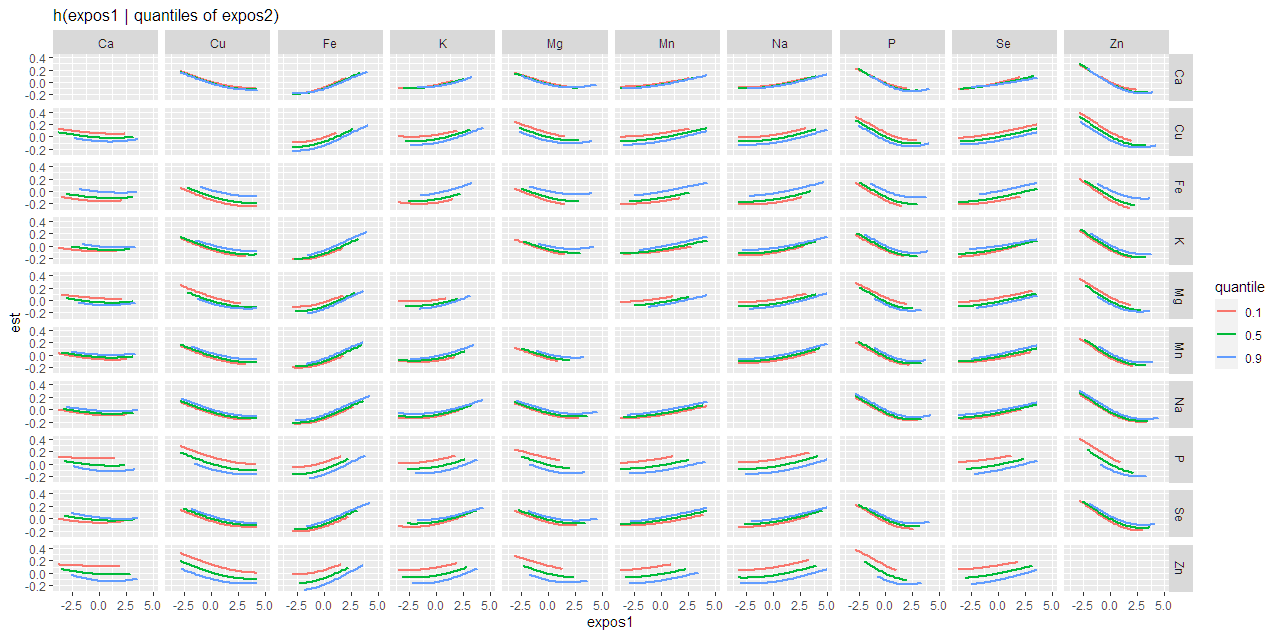


**Figure S2**. Dose-response relationship curves for mixed-exposure first-order interactions
